# International Foot and Ankle Biomechanics Community (i-FAB): past, present and beyond

**DOI:** 10.1186/1757-1146-2-19

**Published:** 2009-06-16

**Authors:** Christopher J Nester, Alberto Leardini, Peter R Cavanagh, Dieter Rosenbaum, Joshua Burns

**Affiliations:** 1 Centre for Rehabilitation and Human Performance Research, School of Health Care Professions, University of Salford, UK; 2 Movement Analysis Laboratory, Istituto Ortopedico Rizzoli, Bologna, Italy; 3 Department of Orthopedics and Sports Medicine, University of Washington, USA; 4 Motion Analysis Laboratory, Orthopaedic Department, University Hospital Muenster, Germany; 5 Discipline of Paediatrics and Child Health, Sydney Medical School, The University of Sydney/Institute for Neuroscience and Muscle Research, The Children's Hospital at Westmead, Sydney, NSW, Australia

## Abstract

The International Foot and Ankle Biomechanics Community (i-FAB) is an international collaborative activity which will have an important impact on the foot and ankle biomechanics community. It was launched on July 2^nd ^2007 at the foot and ankle session of the International Society of Biomechanics (ISB) meeting in Taipei, Taiwan. i-FAB is driven by the desire to improve our understanding of foot and ankle biomechanics as it applies to health, disease, and the design, development and evaluation of foot and ankle surgery, and interventions such as footwear, insoles and surfaces.

## Commentary

i-FAB activities will seek to enable more effective approaches to researching the foot and ankle, accelerate our ability to address the unique challenges the foot and ankle poses for biomechanics researchers and research users, and foster seamless activities between researchers and consumers. The specific objectives of i-FAB are to:

(i) Increase the profile of foot and ankle biomechanics research within academic, clinical and industry communities

(ii) Promote the value of foot and ankle biomechanics research to research users (industry and clinical communities)

(iii) Develop a coordinated approach to addressing the challenges of experimental and computational biomechanics of the foot and ankle

(iv) Enable better coordination of multidisciplinary research

(v) Enable more effective coordination of foot and ankle biomechanics research between groups in different countries

A Steering Group has been formed to guide the initial development of the i-FAB community and its activities. The Steering Group seeks to construct a framework for developing i-FAB to meet the needs of the foot and ankle biomechanics community and welcomes input and direction from members.

In September 2008, we had our first congress at Istituto Ortopedico Rizzoli, Bologna, Italy, chaired by Dr. Alberto Leardini. A mix of 150 lectures, posters and workshops were presented to 170 delegates from 30 different countries. Highlights included a live operation of a total ankle replacement by Professor Sandro Giannini, hands-on multi-segment foot kinematics demonstration by Professor Maria Grazia Benedetti, and keynotes lectures by Professor Harold Kitaoka (Advances in biomechanics of posterior tibial tendon dysfunction and flatfoot deformity), Professor George Arangio (Sailing charted seas: biomechanics and the orthopaedic surgeon), Dr. Mario Lafortune (The role of research in the development of athletic footwear), Professor Peter Cavanagh (Foot complications of diabetes), Dr. Sorin Siegler (Advances in image-based biomechanics of the human ankle) and Dr. Marco Viceconti (Multiscale modelling and team science: the future of orthopaedic biomechanics).

**Figure 1:**
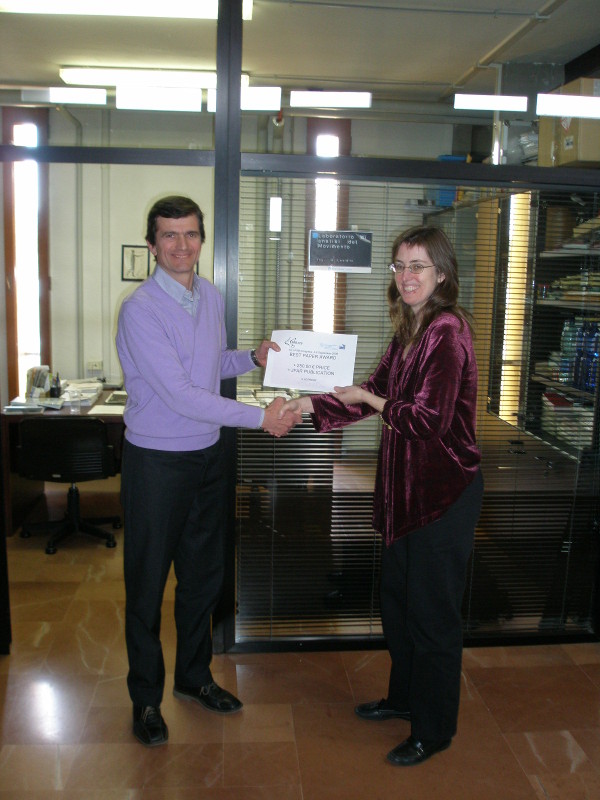
**Dr Frances Sheehan receiving her best paper award from Dr Alberto Leardini**.

The Best Paper of the Congress was awarded to Dr. Frances Sheehan from the Physical Disabilities Branch of the National Institutes of Health, Bethesda, MD, USA for her paper 'Direct in vivo quantification of the 3D talocrural and subtalar finite helical axes' about a revolutionary non-invasive technique for quantifying the 3D *in vivo *axes of the subtalar and talocrural joints using functional MRI during foot plantarflexion and dorsiflexion [1]. Dr. Sheehan was thrilled to be awarded i-FAB's first congress award by Dr. Leardini (see Figure 1). All abstracts have been published as a supplement in the *Journal of Foot and Ankle Research *[2] and can be freely downloaded from the website.

Planning for three i-FAB activities is presently underway: website, 2010 congress, and e-communications. The website and e-communications are the start of a global "collaborative workspace" in which all members of the foot and ankle biomechanics community can engage. This collaborative workspace will harness the most recent IT developments for supporting the organisation and coordination of activities across traditional disciplinary boundaries and between groups who are geographically distributed. We are pleased to announce that the 2010 i-FAB congress will be at The University of Washington in Seattle between September 16^th ^to 18^th ^2010. We anticipate an outstanding program covering both basic and clinical aspects of foot and ankle biomechanics together with a social program that will exploit the resources of the Pacific Northwest.

One exciting goal of i-FAB is to promote and advance research in the field of foot and ankle biomechanics by providing up-to-date consensus based standards for the study and description of foot and ankle kinematics. We have assembled a committee to provide standardised quantitative morphological databases and periodic reviews of the state-of-the-art in foot and ankle kinematic research.

i-FAB welcomes members from every community related to foot and ankle biomechanics, from academics, physicians, podiatrists, surgeons, and health professionals, to members of the footwear, orthosis, surgery and related industries. i-FAB has an open philosophy and connecting people across traditional disciplinary boundaries is one of its key objectives. Membership is presently free. Further details regarding i-FAB can be found on our website [3].
